# Sorafenib-induced defective autophagy promotes cell death by necroptosis

**DOI:** 10.18632/oncotarget.5797

**Published:** 2015-09-22

**Authors:** Pedram Kharaziha, Dimitris Chioureas, George Baltatzis, Pedro Fonseca, Patricia Rodriguez, Vladimir Gogvadze, Lena Lennartsson, Ann-Charlotte Björklund, Boris Zhivotovsky, Dan Grandér, Lars Egevad, Sten Nilsson, Theocharis Panaretakis

**Affiliations:** ^1^ Department of Oncology-Pathology, Cancer Centrum Karolinska, Karolinska Institutet and University Hospital, Stockholm, Sweden; ^2^ Department of Medicine, School of Health Sciences, University of Athens, Athens, Greece; ^3^ Department of Medical Biochemistry and Biophysics, Karolinska Institutet, Stockholm, Sweden; ^4^ Institute of Environmental Medicine, Division of Toxicology, Karolinska Institutet, Stockholm, Sweden

**Keywords:** prostate cancer, tyrosine kinase inhibitor, autophagy, Atg5, necroptosis

## Abstract

Autophagy is one of the main cytoprotective mechanisms that cancer cells deploy to withstand the cytotoxic stress and survive the lethal damage induced by anti-cancer drugs. However, under specific conditions, autophagy may, directly or indirectly, induce cell death. In our study, treatment of the Atg5-deficient DU145 prostate cancer cells, with the multi-tyrosine kinase inhibitor, sorafenib, induces mitochondrial damage, autophagy and cell death. Molecular inhibition of autophagy by silencing ULK1 and Beclin1 rescues DU145 cells from cell death indicating that, in this setting, autophagy promotes cell death. Re-expression of Atg5 restores the lipidation of LC3 and rescues DU145 and MEF atg5−/− cells from sorafenib-induced cell death. Despite the lack of Atg5 expression and LC3 lipidation, DU145 cells form autophagosomes as demonstrated by transmission and immuno-electron microscopy, and the formation of LC3 positive foci. However, the lack of cellular content in the autophagosomes, the accumulation of long-lived proteins, the presence of GFP-RFP-LC3 positive foci and the accumulated p62 protein levels indicate that these autophagosomes may not be fully functional. DU145 cells treated with sorafenib undergo a caspase-independent cell death that is inhibited by the RIPK1 inhibitor, necrostatin-1. Furthermore, treatment with sorafenib induces the interaction of RIPK1 with p62, as demonstrated by immunoprecipitation and a proximity ligation assay. Silencing of p62 decreases the RIPK1 protein levels and renders necrostatin-1 ineffective in blocking sorafenib-induced cell death. In summary, the formation of Atg5-deficient autophagosomes in response to sorafenib promotes the interaction of p62 with RIPK leading to cell death by necroptosis.

## INTRODUCTION

Prostate cancer (PCa) is the most common malignancy in men and the metastatic, castration resistant prostate cancer (CRPC) is the second most frequent cause of cancer related deaths in the western world [1]. Currently, the second line therapeutic modalities for CRPC do not offer significant survival benefits but rather have a palliative role. Several novel cancer therapeutics against CRPC have been lately introduced into the clinic, including single or multi-tyrosine kinase (TKI) inhibitors.

Receptor and non-receptor tyrosine kinases (TK) regulate multiple signal transduction pathways that promote growth, resistance to cell death and metastasis of most cancers including prostate cancer [2]. Progression from PCa to CRPC has been associated with the constitutive activation of receptor TKs (e.g. platelet-derived growth factor receptor β (PDGFRβ), fibroblast growth factor receptor (FGFR), vascular endothelial growth factor receptor (VEGFR)) and as non-receptor TK (e.g. Src) [3]. Hence, several targeted TKI such as sunitinib, erlotinib, dasatinib and sorafenib have been developed and tested in the clinic for their efficacy against prostate cancer [4-6].

Sorafenib, a type II TKI, targets the Raf kinases including Raf-1 and b-Raf, as well as VEGFR-2 and -3, PDGFR-β, Flt-3 and c-KIT, most of which are known to be activated in CRPC [5]. Sorafenib has been introduced in several prostate cancer clinical trials and the initial data coming out indicate that in some patients, sorafenib treatment may have beneficial effects but further follow-ups are required [7]. Apart from inhibiting TK, sorafenib, at clinically relevant concentrations, has been shown to severely disrupt mitochondrial respiration and function, by directly inhibiting complex II, III and V of the electron transport chain [8].

A frequent effect observed in the clinic and in experimental cancer models treated with tyrosine kinase inhibitors is the induction of autophagy [9-13]. Autophagy is regulated by well characterised and orchestrated sequence of molecular events that lead to the formation of the autophagosomes, vesicles that engulf the cytoplasmic content targeted for degradation by the lysosomes [14]. It is a cytoprotective, physiological process that maintains homeostasis by relieving the cell from cytotoxic, damaged organelles as well as support survival during conditions of metabolic stress [15, 16]. In the cancer setting, autophagy has a dual role both of which aim to protect the cancer cells. In the early stages of tumorigenesis, autophagy plays a tumor suppressive role by eliminating intracellular insults that may induce DNA damage, genetic instability and promote irregular cell growth [17]. Thus, frequently, autophagy is temporarily inhibited to allow for cancer cell growth. In the later stages of tumor growth and in metastasis, autophagy plays a tumor promoting role providing cancer cells with the necessary nutrients, intracellular and extracellular defences to survive, adapt and disseminate [17].

Even though in most cases autophagy plays a cytoprotective role, there are several experimental evidences pointing towards to being a mechanism of cell death in a cell type and stress-specific manner [18-20]. Autosis was recently introduced as a mode of autophagic cell death that is mediated by the inhibition of the Na+, K+-ATPase pump and has unique morphological features [20]. Alternatively, autophagy may employ the necroptosis machinery to induce cell death in Rhabdomyosarcoma cell lines, upon treatment with the anti-apoptotic Bcl-2 antagonist Obatoclax [21]. This newly discovered cross-talk between autophagy and necroptosis was also demonstrated in renal cell carcinoma cells where the induction of autophagy by rapamycin suppresses the RIPK-dependent necroptosis by facilitating the degradation of RIPK1 and RIPK3 [22].

Necroptosis is a caspase-independent form of regulated cell death [23, 24]. It is induced by a wide variety of stimuli (e.g. cell death ligands, pathogen recognition receptors, viral RNA sensors, DNA damage and hypoxia) which converge at the formation of a supramolecular complex called the necrosome [25]. Formation of the necrosome leads to the activation of the receptor-interacting protein kinase 1 (RIPK1), its substrate RIPK3 and the downstream executioner of necroptosis, Mixed Lineage Kinase Like (MLKL) [23]. Several experimental evidence with RIPK1, RIPK3 and MLKL knockout mice as well as clinical studies have demonstrated that necroptosis plays an important role in a variety of several pathological conditions including cancer, myocardial infarction and stroke, ischemia reperfusion injury and atherosclerosis [26, 27].

In the present study, we provide evidence that Atg5-deficient DU145 prostate cancer cells undergo caspase-independent, autophagic cell death in response to sorafenib. This mode of cell death can be prevented by either the re-expression of Atg5 or the molecular inhibition of key autophagy regulators such as ULK1 or Beclin1. Furthermore we show that induction of autophagy leads to the activation of necroptosis in a p62-dependent manner and chemical or molecular inhibition of RIPK1 renders the cells resistant to sorafenib.

## RESULTS

### Sorafenib induces autophagy-dependent cell death in DU145 cells

**Figure 1:**
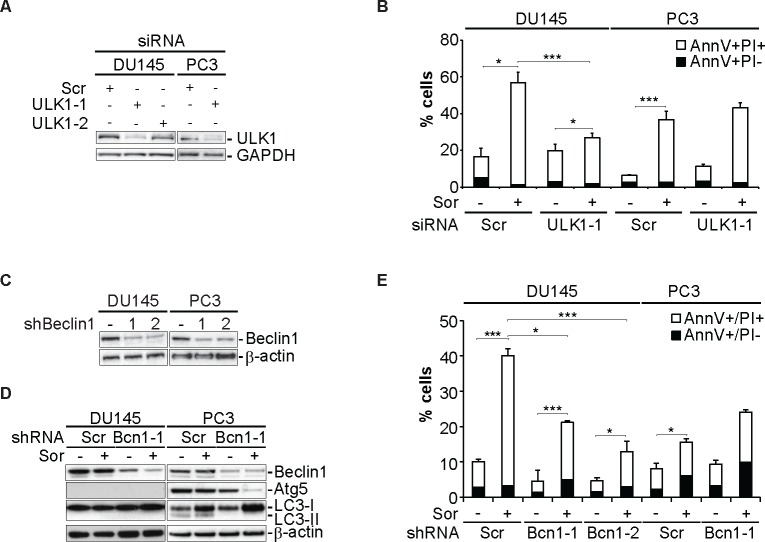
Sorafenib induces autophagy-dependent cell death in DU145 cells **A.** Western blot analysis of DU145 and PC3 cells transiently transfected with siScr or siULK1 and probed for ULK1 and GAPDH; **B.** Quantitative analysis of Annexin V/PI positive DU145 and PC3 cells transiently transfected with siScr or siULK1 (means ± SD, *n* = 3, *<0.05, ***<0.005); **C.** Western blot analysis for the indicated proteins of DU145 and PC3 cells stably transfected with either shScramble (shScr) or two Beclin1 shRNA constructs (shBcn1-1 and shBecn1-2); **D.** Western blot analysis for the indicated proteins of DU145 and PC3 cells stably transfected with either shScr or shBcn1-1 and probed for the indicated proteins; **E.** Quantitative analysis of Annexin V/PI positive of either shScr or shBcn1 cells treated with 20 μM Sor for 24h (means ± SD, *n* = 3, *<0.05, ***<0.005).

We have previously shown that sorafenib (Sor) induces cell death in 22Rv1 and PC3 prostate cancer (PCa) cell lines [28]. The cytotoxic efficacy of Sor is increased when autophagy is inhibited by chemical or molecular means [28]. In contrast to these two PCa cell lines, Sor-induced-cell death in DU145 cells was potentiated upon inhibition of the autophagic machinery. Silencing of ULK1, an important regulator of the autophagy-initiation step, with two specific siRNAs led to a significant decrease of Sor-induced cell death in DU145 cells whereas it did not have any effect in PC3 cells (Figure 1A and 1B). We examined further the role of autophagy in Sorafenib-induced cell death by utilising two different shRNA constructs against Beclin1 (Figure 1C and 1D). Stable transfection with these Beclin1 shRNA constructs also protected DU145 cells from Sor-induced cell death whereas it potentiated cell death in PC3 cells (Figure 1E). These data indicate that the autophagic machinery is involved in Sor-induced cell death.

### Sorafenib induces the formation of LC3 positive autophagosomes in the Atg5 deficient, DU145 cells

It was previously shown that Sor induces mitochondrial damage by directly inhibiting complex II, III and V of the respiratory chain in the mitochondria, leading to severe mitochondrial damage and depolarisation in isolated mitochondria and in liver cancer stem cells [8, 29]. In agreement with these observations, we found, by transmission electron microscopy and confocal microscopy, that treatment of DU145 cells with 20μM Sor resulted in extensive mitochondrial damage (Supplementary figure 1A and 1B). Treatment with Sor also led to an inhibition of mitochondrial respiration already at 4h and a decrease in intracellular ATP levels (Supplementary figure 1C and 1D). Cell death analysis by flow cytometry of DU145 cells labelled with Annexin V (cell death marker) and TMRE (functional mitochondria marker) demonstrated a rapid decrease in mitochondrial membrane potential at 4h followed by Annexin V positive staining (Supplementary figure 1E and 1F).

**Figure 2:**
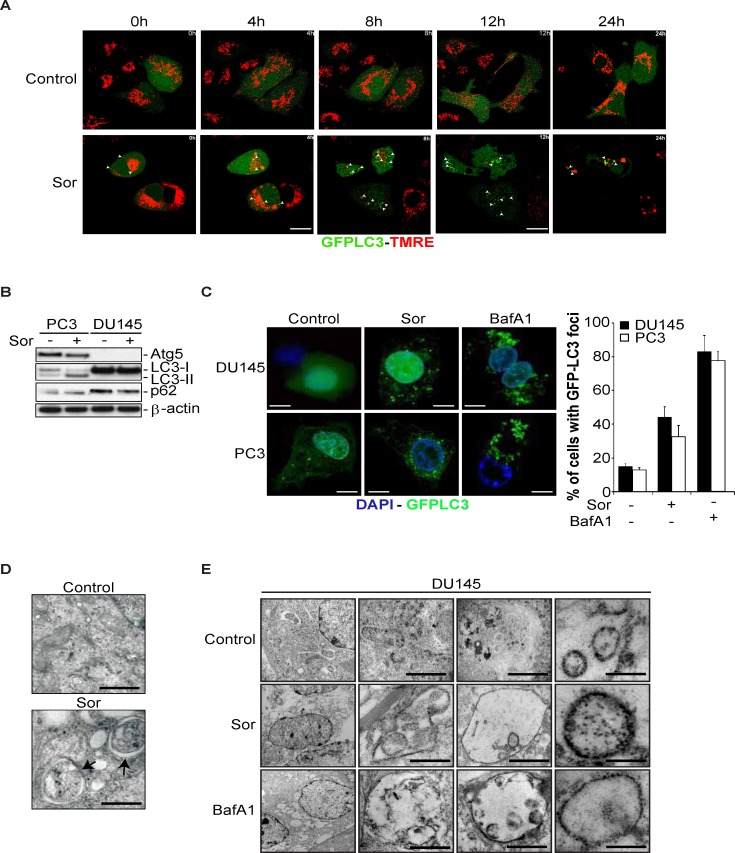
Sorafenib induces the formation of Atg5-independent autophagosomes in DU145 cells **A.** Time lapse confocal microscopy images of DU145 cells stably transfected with GFP-LC3 and stained with TMRE followed by treatment with 20 μM Sor for the indicated time points (Scale bar: 2 μm); **B.** Western blot of the indicated proteins of DU145 and PC3 cells treated with 20 μM Sor for 24h; **C.** Confocal microscopy imaging and quantification of DU145 and PC3 cells stably transfected with GFP-LC3 and treated with 20 μM Sor or 10 nM BafA1 for 24h; **D.** Transmission electron microscopy of DU145 cells treated with 20 μM Sor for 24h; **E.** Immuno-electron microscopy against LC3 in DU145 cells treated with 20 μM Sor or 10 nM BafA1 for 24h (Scale bar: 500 nm).

It is known that autophagy is one of the main mechanisms of removing damaged organelles such as mitochondria (i.e. mitophagy) from the cells [30]. In an attempt to correlate the Sor-induced mitochondrial dysfunction with autophagy, we performed a time lapse confocal microscopy experiment. DU145 cells stably transfected with GFP-LC3 were stained with TMRE. After 4h of treatment, mitochondrial depolarisation was evident and was followed by the appearance of multiple GFP-LC3 foci by 8h up to 24h after Sor treatment (Figure 2A).

The detection of these GFP-LC3 foci was surprising since it has been shown that DU145 cells do not undergo autophagy in response to starvation and Valproic acid treatment due to the lack of *Atg5* expression [31]. This is due to the expression of alternative *Atg5* transcripts that lack one or two exons, leading to the premature termination of *Atg5* protein translation. We confirmed the lack of *Atg5* expression, in our experimental setting, the lack of LC3 lipidation as well as an observed accumulation of p62 protein levels compared to PC3 cells, none of which changed upon treatment with Sor (Figure 2B).

Treatment of DU145 cells with Sor revealed intracellular structures characteristic of autophagosomes as judged by confocal microscopy images and time lapse microscopy of GFP-LC3 transfected cells (Figure 2C and time-lapse videos 1 and 2). Similar data were obtained by confocal fluorescent microscopy for stainings of the endogenous LC3 and p62 proteins (Supplementary Figure 2). Furthermore, GFP-LC3 positive foci, characteristic of autophagic vesicles were also detected in DU145 cells treated with the late autophagy inhibitor bafilomycin A1 (BafA1), (Figure 2C). Interestingly, these GFP-LC3 foci were similar to those observed in PC3 cells that express Atg5. The observed induction of GFP-LC3 positive foci upon treatment with Sor was also evident in MEF cells that are deficient in Atg5. Importantly, reconstitution of Atg5 expression in MEF *Atg5−/−* cells led to a decrease of GFP-LC3 autophagic foci to levels similar to wt MEF cells (Supplementary Figure 3A, 3B and 3C).

We examined the presence of LC3 by immunogold-electron microscopy in the observed, by transmission electron microscopy, autophagosomal structures (Figure 2D and 2E). We found that LC3 was lining up the membrane of the autophagosomes in DU145 cells treated with either Sor or BafA1.

Since we could not monitor the autophagic flux by one of the classical methods, i.e. by measuring the levels of LC3 lipidation, we stably transfected the cells with the tandem GFP-RFP-LC3 construct. This assay is based on the different pH stability of EGFP and mRFP fluorescent proteins with yellow foci indicating the presence of autophagosomes and the presence of red foci the presence of autophagolysosomes. We found that in DU145 cells, GFP-RFP-LC3 (yellow) foci formed upon treatment with Sor or BafA1 but very few red foci, indicative, perhaps of their inefficient degradation by lysosomes (Supplementary Figure 4A and 4B). These data were in contrast to PC3 cells in which many foci were RFP positive indicative of the formation of autophagolysosomes and the subsequent quenching of EGFP due to low pH.

We also utilised the long-lived protein degradation assay to assess the functionality and efficacy of the autophagic process. We have utilised a recently published, fluorescent variant of the classical, radioactive long-lived protein degradation method [32]. We measured the levels of the long-lived proteins in DU145 cells by flow cytometry and visualised them by confocal microscopy (Supplementary Figure 5A and 5B). We found that treatment with Sor increased the amount of fluorescently labelled long-lived proteins compared to control, suggesting that treatment with Sor inhibits their degradation by autophagolysosomes. Treatment with BafA1 led to a bigger increase in the levels of the long-lived proteins (Supplementary Figure 5A and 5B). To further address the effect of Sor on the autophagic machinery we used DU145 cells stably transfected with an shRNA against Beclin1 or transiently with siRNA against ULK1. In both settings, knockdown of these proteins led to the accumulation of long-lived proteins in the control cells and treatment with Sor did not further increase the levels of long-lived proteins (Supplementary Figure 5C and 5D). These data indicate that although Sor induces the formation of autophagosomal structures in an Atg5-independent manner, they may be inactive.

### Re-constitution of ATG5 expression rescues DU145 cells from Sor-induced cell death

**Figure 3:**
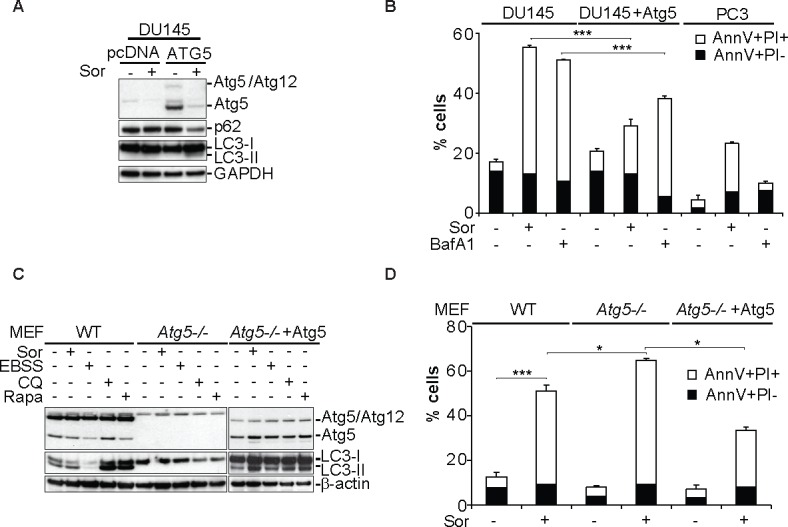
Expression of Atg5 rescues DU145 cells from Sor-induced cell death **A.** Western blot analysis of the indicated proteins in DU145 cells transiently transfected with a pcDNA plasmid (vehicle) or Atg5 followed by treatment with 20 μM Sor for 24h; **B.** Quantitative analysis of Annexin V/PI positive DU145 and PC3 cells transiently transfected with either mock plasmid or Atg5 and treated with 10 nM BafA1 followed by 20 μM Sor for 24h, (means ± SD, *n* = 3, ***<0.005); **C.** Western blot analysis for the indicated proteins of MEF WT, *Atg5*−/− and Atg5 reconstituted cells treated with 20 μM Sor, or EBSS, or 50 μM Chloroquine (CQ) or 1μM Rapamycin (Rapa) for 24h; **D.** Quantitative analysis of Annexin V/PI positive cells of either WT, *Atg5−/−* or *Atg5−/−* transiently transfected with Atg5 followed by treatment with 20 μM Sor for 24h (means ± SD, *n* ≥ 3, *<0.05, ***<0.005).

The significance of the Atg5 loss of expression in Sor-induced cell death was examined in DU145 cells by transiently re-expressing Atg5 (Figure 3A). Reconstitution of Atg5 expression rescued DU145 cells from Sor-induced cell death (Figure 3B). These data were recapitulated in *Atg5−/−* MEFs which, as observed with DU145 cells, were more sensitive to Sor compared to WT cells and ectopic expression of Atg5 leads to the re-constitution of LC3 lipidation and a decrease in Sor-induced cell death (Figure 3C and 3D). Collectively these data further demonstrate that Sor-induced autophagy in Atg5 deficient cells is cytotoxic and restoration of Atg5 expression protects the cells from Sor-induced cell death.

### Sorafenib-induced cell death in DU145 cells is executed by necroptosis

To decipher the mode of cell death induced in DU145 cells by Sor, we utilised the pancaspase inhibitor zVAD.fmk and the caspase-9 inhibitor LEHD.fmk. We found that Sor-induced cell death was not blocked by either of these two inhibitors, in contrast to LNCaP cells which were rescued from cell death (Supplementary Figure 6A). We could not detect caspase-3 activity in DU145 cells treated with Sor nor cleavage of the caspase substrate PARP indicating that caspases are not activated in response to Sor (Supplementary Figure 6B and 6C). In addition, there were no changes in Bak, Bax, Bcl-xL and AIF protein levels, nor any activation of Bak and Bax detected, all of which indicate that Sor induced a caspase-independent cell death in DU145 cells (Supplementary Figure 6C, 6D and data not shown). This is in contrast to 22Rv1 and PC3 PCa cells which undergo caspase-dependent cell death in response to Sor [33].

**Figure 4:**
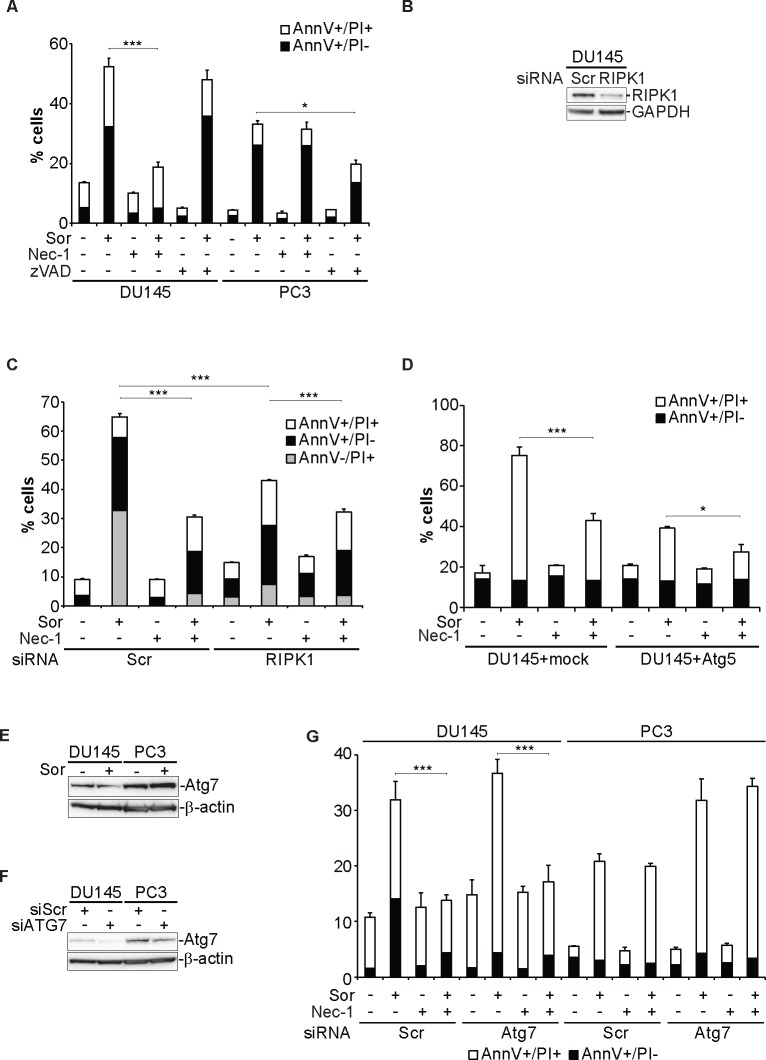
Sorafenib-induced cell death in DU145 cells is executed by necroptosis **A.** Quantitative analysis of Annexin V/PI positive DU145 and PC3 cells pre-treated with either 10 μM zVAD.fmk or 50 μM Nec-1 followed by 20 μM Sor for 48h, (means ± SD, *n* = 3 *<0.05, ***<0.005); **B.** Western blot analysis for the indicated proteins of DU145 transiently transfected with Scr or RIPK1 siRNA; **C.** Quantitative analysis of Annexin V/PI positive DU145 cells transfected either with Scr or RIPK1 siRNA pool, pre-treated with 50 μM Nec-1 followed by 20 μM Sor for 48h, (means ± SD, *n* = 3, ***<0.005); **D.** Quantitative analysis of Annexin V/PI positive DU145 cells transfected either with a mock plasmid or Atg5, pre-treated with 50 μM Nec-1 followed by 20 μM Sor for 48h, (means ± SD, *n* ≥ 3, *<0.05, ***<0.005).

Having observed that Sor-induced cell death is caspase-independent we examined whether necroptosis is activated. Treatment of DU145 cells with the RIPK1 inhibitor, Necrostatin 1 (Nec-1), inhibited Sor-induced cell death but had no effect in PC3 cells (Figure 4A) [33]. Similarly, reconstitution of Atg5 expression in *Atg5−/−* MEFs under the control of the doxycycline-regulated system further demonstrated that the observed increased death was due to the loss of Atg5 expression and that this death can be partially protected by Nec-1 (Supplementary Figure 7A and 7B). Furthermore, Nec-1 partially protected PC3 cells from Sor-induced cell death, in which *Atg5* was knocked down with an siRNA (Supplementary Figure 7C). Transient knockdown of RIPK1, with a smartpool of siRNAs, inhibited Sor-induced cell death and the addition of Nec-1 further promoted the survival of DU145 cells (Figure 4B and 4C). Importantly, ectopic expression of Atg5 in DU145 cells protected from Sor-induced cell death and co-treatment with Nec-1 further inhibited cell death (Figure 4D).

Another major molecular player in the autophagic cascade, mediating the lipidation of LC3 is Atg7. We examined the total protein levels of Atg7 in DU145 and PC3 cells. DU145 cells have lower Atg7 protein levels than PC3 cells and Sor treatment does not have any effect on them (Figure 4E). Silencing of Atg7 in DU145 had no effect in Sor inducing cell death which was inhibited by Nec-1 (Figure 4F and 4G). In contrast, PC3 cells with lower Atg7 levels were more sensitive to Sor than the siScr transfected cells and this cell death was not affected by Nec-1 (Figure 4F and 4G).

We then examined whether the inhibition of Sor-induced cell death by Nec-1 was due to an indirect effect on the autophagic machinery. The effect of Nec-1 on the degradation of long-lived proteins was examined by flow cytometry (Supplementary Figure 8A). We did not observe any effects of Nec-1 either alone or in combination with Sor. We also examined whether Nec-1 has any effect on the formation of the GFP-LC3 positive foci induced by Sor in DU145 cells. Nec-1 alone did not induce GFP-LC3 foci and the combination of Nec-1 with Sor did not change the number of cells with GFP-LC3 positive foci compared to Sor alone (Supplementary Figure 8B and 8C). In summary these data indicate that Sor-induced cell death in DU145 cells is due to the loss of Atg5 expression and is mediated by necroptosis.

### p62 is required for Sorafenib induced necroptosis in DU145 cells

**Figure 5:**
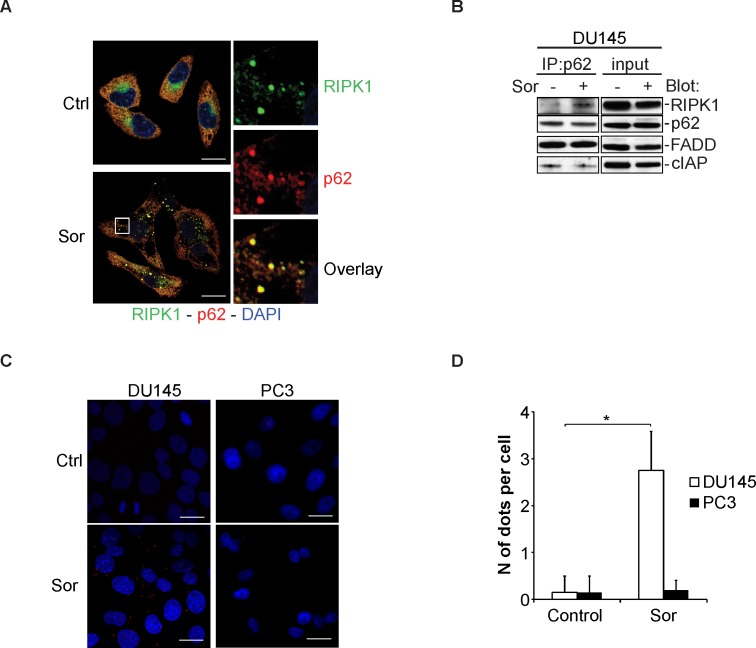
Sorafenib induces the interaction between p62 and RIPK1 **A.** Representative confocal microscopy images of DU145 cells stained for p62 and RIPK1 after treatment with 20μM Sor for 24h (scale bar: 2 μm); **B.** Western blot analysis for the indicated proteins following immunoprecipitation with p62 after treatment with 20μM Sor for 24h; **C.** Control and 20 μM Sor-treated DU145 and PC3 cells for 24h were stained with anti-p62 and anti-RIPK1 antibodies and detected using the proximity ligation assay. Compilation of ten Z-stack images captured by a confocal microscope is shown here (Scale bar: 2 μm). **D.** Quantification of dots per cell in DU145 and PC3 cells upon treatment with 20 μM Sor for 24h by using the blobfinder software (*n* = 3, *<0.05).

The p62 is a ubiquitin-binding, scaffold protein that plays a major role in delivering cytoplasmic cargo targeted for degradation by autophagy. It has also been shown that p62 acts as a scaffold protein for the activation of RIPK1 and the NF-kB pathway [34]. In DU145, there are accumulated protein levels of p62, a cellular response usually associated with defective autophagic degradation of p62 bound substrates. We hypothesised that p62 may act as a signalling platform for the activation of RIPK1 and RIPK3 in response to Sor. Immunocytochemical staining of DU145 cells for p62 and RIPK1 revealed that, upon treatment with Sor, there is an increased co-localisation between these two proteins (Figure 5A). Co-immunoprecipitation and the proximity ligation assay (PLA) experiments further demonstrated that Sor promotes the interaction between RIPK1 and p62 (Figure 5B, 5C and 5D). Importantly, this increased interaction was not observed in PC3 cells as examined by confocal microscopy (Supplementary Figure 9).

**Figure 6:**
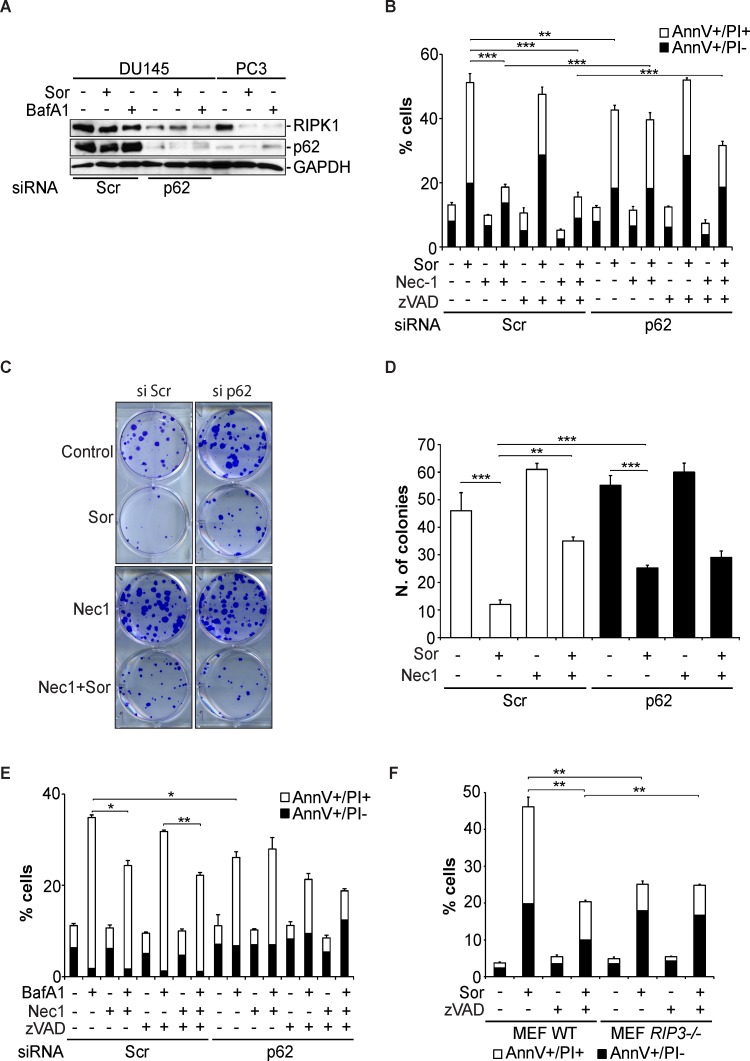
Sorafenib-induced necroptosis in DU145 cells is p62 and RIPK1-dependent **A.** Western blot analysis of the indicated proteins expressed in DU145 cells transfected with either scramble siRNA (siScr) or p62 siRNA (sip62) followed by pre-treatment with either 20 μM Sor or 10 nM BafA1 for 48h; **B.** Quantitative analysis of Annexin V/PI positive DU145 cells transfected with either siScr or sip62 followed by pre-treatment with 50 μM Nec-1 followed by 20 μM Sor for 48h, (means ± SD, *n* ≥ 3, **<0.01, ***<0.005); **C.** Clonogenic survival assay of DU145 cells transfected with either siScr or sip62, pre-treated with 50 μM Nec-1 followed by 20 μM Sor for 16h and cultured for 10 days; **D.** Quantification of the colonies found in **C.**, (means ± SD, *n* ≥ 3, **<0.01, ***<0.005); **E.** Quantitative analysis of Annexin V/PI positive DU145 cells transfected with either siScr or sip62 followed by pre-treatment with 50 μM Nec-1 followed by 10 nM BafA1 for 48h, (means ± SD, *n* ≥ 3, **<0.01, ***<0.005); **F.** Quantitative analysis of Annexin V/PI positive MEF wt or MEF RIPK3−/− cells treated with 20 μM Sor for 24h, (means ± SD, *n* ≥ 3, *<0.05, ***<0.005).

Having established that p62 interacts with components of the necroptosome, we examined the role of this interaction in Sor-induced autophagic cell death. DU145 cells transiently transfected with control siRNA (siScr), treatment with Sor did not change the intracellular levels of RIPK1 (Figure 6A). In contrast, treatment of PC3 cells with Sor decreased RIPK1 levels. Importantly, knocking down p62 with a specific siRNA in DU145 cells decreased the basal levels of RIPK1 protein suggesting that p62 protects RIPK1 from degradation (Figure 6A). Furthermore, silencing of p62 decreased the basal levels of RIPK1 in DU145 cells, rendering Nec-1 ineffective in blocking Sor-induced cell death (Figure 6B). To further demonstrate the importance of p62 and RIPK1 in Sor-induced necroptosis we performed clonogenic assays in DU145 cells. The colonies formed in Sor-treated DU145 cells transfected with siScr increased in the presence of Nec-1. Knocking down of p62 significantly increased the clonogenic survival of Sor-treated DU145 cells and Nec-1 did not have any further effect (Figure 6C and 6D). The importance of p62 for autophagy-mediated necroptosis was recapitulated with BafA1, where Nec-1 could no longer block BafA1-induced caspase-independent cell death in p62 depleted cells (Figure 6E).

To further investigate the involvement of necroptosis in Sor-induced cell death we utilised *RIPK3−/−* MEF cells, a major component and regulator of necroptosis. Wild type MEF cells were sensitive to Sor. In contrast, *RIPK3−/−* MEF cells were less sensitive to Sor and Nec-1 had no effect on the levels of cell death (Figure 6F).

## DISCUSSION

Sorafenib, a type II TK inhibitor, is an attractive TKI for the treatment of CRPC since it targets multiple tyrosine kinase including VEGFR, PDGFR-β and Src. In a previous study, we described that sorafenib induces caspase-dependent cell death in 22Rv1 and PC3 cell lines and inhibition of autophagy potentiates the efficacy of this TKI [28, 33]. In this study we provide evidence that DU145 cells undergo caspase-independent cell death in response to Sor and that this death is mediated by autophagy.

It is known that apart from targeting multiple tyrosine kinases, sorafenib directly impairs mitochondrial function by acting as an uncoupler and inhibitor of state II respiration [8]. Indeed, we found that sorafenib inhibits mitochondrial respiration, decreases mitochondrial membrane potential and the intracellular levels of ATP within 4h of sorafenib treatment. Autophagy is the main mechanism of protection against such intracellular damage acting to remove the damaged mitochondria by a process called mitophagy. We have found by time-lapse confocal microscopy that the mitochondrial depolarisation precedes the formation of GFP-LC3 foci which later on accumulate and are sustained till the extensive vacuolisation and death of the cell.

The formation of the GFP-LC3 foci is surprising since these cells are deficient in Atg5 and the ensuing LC3 lipidation. It was previously demonstrated that mouse cells lacking Atg5 or Atg7 can still form autophagosomes/autophagolysosomes and perform autophagy-mediated protein degradation when subjected to stressors such as etoposide [35]. This Atg5/Atg7-independent autophagy is not regulated by Ulk1 and Beclin1 but by Rab9, the GTPase that drives the formation of the autophagosomes in the absence of LC3. Our data do not support this model since we have identified the presence of LC3 positive foci in structures resembling autophagosomes by several means: i) confocal microscopy against GFP-LC3 as well as the endogenous levels of LC3; ii) GFP-RFP-LC3 positive foci; and iii) immunogold electron microscopy for LC3. The presence of autophagic vacuoles in the absence of LC3 lipidation has been reported in the *Atg5−/−* purkinje cells which accumulate aberrant autophagosome-like double membrane structures prior to their death [36]. However, these autophagosome-like structures are not functional. In our experimental setting, we have evidence that although Sor induces the formation of these autophagosomal-like structures, they are not functional as judged by i) the sustained high levels p62 protein levels; ii) the lack of content in the immuno-electron microscopy images; and iii) the increase in the amount of long-lived proteins in response to Sor. Thus, we believe that DU145 cells in an attempt to alleviate the mitochondrial and other intracellular damages inflicted by Sor, evoke an autophagic response which due to the lack of Atg5 is defective or inefficient. In an alternative setting, it may be that Sor has a direct effect on the lysosomes rendering them inactive which could explain the accumulation of GFP-RFP-LC3 positive vacuoles.

The reconstitution of Atg5 expression in DU145 cells rescued the cells from Sor-induced cell death in a similar manner as ULK-1 and Beclin1 silencing. Similar findings we had with *Atg5−/−* MEF and the re-expression of Atg5 which also rescued them from Sor-induced caspase-independent cell death. DU145 cells may have adopted to survive under basal stress conditions and organellar turnover by activating alternative modes of autophagy such as Atg5/Atg7 independent autophagy, micro-autophagy or chaperone-mediated autophagy. However when confronted with the massive mitochondrial damage as that induced by Sor they activate the classical autophagic pathway which is inefficient and defective due to the lack of Atg5 and LC3 lipidation.

**Figure 7:**
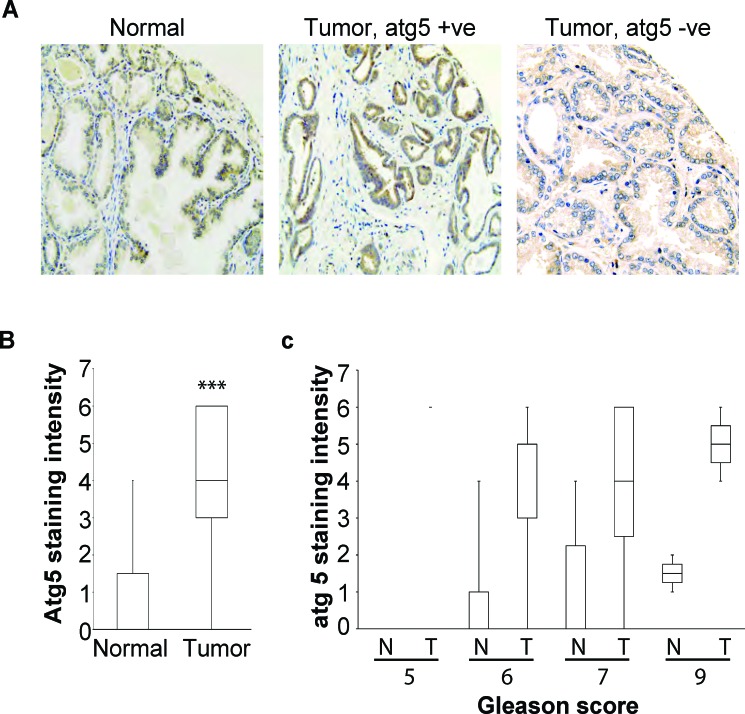
**A.** Representative tissue sample images of Atg5 immunohistochemical stainings from a healthy individual and a patient with prostate cancer. Each tissue core is 0.6 mm in diameter; **B.** Tissue samples from healthy individuals (N) and prostate cancer patients (T) were stained for Atg5 and the mean level of staining intensity was quantified and plotted (***<0.005); **C.** Quantitative analysis of the intensity of Atg5 tissue staining for each of the 28 samples included in the tissue microarray indicating the Gleason score for each sample.

Autophagy has a dual role in tumor initiation, progression and metastasis. It has been shown that autophagy may act as a tumor suppressive mechanism at the early stages of tumorigenesis and as a tumor promoting mechanism at the stages of tumor growth and metastasis. In both scenarios, however, autophagy acts as homeostatic, cytoprotective mechanism and this is exploited by cancer cells to their advantage. We examined the frequency of Atg5 loss in prostate cancer patients by performing a tissue micro array and stained for intracellular Atg5 levels by immunohistochemistry. We found that in the majority of the clinical samples there is high expression of Atg5 and there was no correlation with the Gleason score (Figure 7). These findings are in agreement with a previous study where Kim et al., also found overexpression of Atg5 in the majority of the prostate cancer patient samples [37]. However, in our study we detected the loss of Atg5 expression in 18% of the CRPC patients. Similar findings where obtained in 21% of gastrointestinal cancer patients demonstrating loss of Atg5 expression [38]. Furthermore, the down-regulation of Atg5 expression has been shown in colorectal cancer and early cutaneous melanoma patients with prognostic and diagnostic implications [39, 40]. The tumor growth promoting benefits of Atg5 or Atg7 loss of expression has also been demonstrated in several experimental models, such as in the BRAF^V600E^ driven lung carcinomas or in the KRAS^G12D^ or driven pancreatic cancer [41, 42]. Our observation that cancer cells that have lost the expression of Atg5 may undergo deficient or inefficient autophagy in response to cytotoxic insults has important clinical implications. Treatment of cancer cells with autophagy inhibitors (e.g. hydroxochloroquine, Lys05 or Vps34 inhibitors) often ameliorates the efficacy of cancer therapy but in cells with deficient autophagy may promote the survival of these cells [43]. However, in a subset of patients where autophagy may be defective, inefficient and may promote cell death, alternative strategies are required, such that will promote and induce autophagy in order to increase the intracellular stress to the point of no return. Thus, this particular subset of patients would benefit from inducers of autophagy as a combination therapy like the mTOR inhibitors (e.g. rapalogues) or anti-apoptotic Bcl-2 antagonists (e.g. ABT737 or obatoclax).

There are several reports indicating that autophagy may promote or act as a cell death mechanism in response to anti-cancer drugs [18, 19, 21]. The mechanisms by which autophagy would promote or mediate caspase-independent cell death are not clearly defined. Interestingly, the interplay between mitophagy and necroptosis has been previously reported [44]. Furthermore, it has been previously shown that autophagy inhibits necroptosis by promoting the degradation of RIPK1 [22]. The recent demonstration of the importance of Atg5 in the activation of the necrosome in response to Obatoclax-induced autophagic cell death provide further evidence of the extensive cross-talk between the autophagic and the necroptosis machinery [21]. In this study we show that necroptosis is the mechanism by which DU145 cells undergoing Atg5-deficient autophagy succumb to sorafenib. Pre-treatment of DU145 cells with necrostatin-1 potently inhibited cell death induced by sorafenib. Furthermore, MEF cells deficient in RIPK3 are less sensitive to sorafenib compared to wt cells and this death is necrostatin-independent. In this setting we propose that Atg5-deficient autophagy allows for the accumulation of p62 protein levels that promote the formation of the necrosome and the activation of the RIPK1/RIPK3 necroptotic cell death pathway. This hypothesis is supported by the data showing that in DU145 cells, the interaction of p62 with RIPK1 is induced upon sorafenib treatment and that knockdown of p62 leads not only to the downregulation of RIPK1 protein levels but also renders DU145 cells insensitive to necrostatin-1. However, further experiments are required to determine the molecular regulation and significance of these findings.

In summary, we provide evidence that defective or inefficient autophagy will lead to the accumulation of p62 protein levels which upon intracellular damage or stress may act as a signalling platform for the activation of necroptosis as a mode of cell death. From a therapeutic point of view, we provide evidence that sorafenib, a drug that is currently on clinical trials for prostate cancer and used for hepatocellular and renal cancers, may kill cancer cells by necroptosis when autophagy is defective or deficient. This finding provides a rationale for screening of patients for an intact autophagic machinery since such characterisation would reveal the most efficient therapeutic modalities in a personalised manner. It follows that treatment of patients that lack or have mutant key autophagy regulators would benefit from novel combination treatments with agents that promote the induction of autophagy such as the rapamycin analogues and the anti-apoptotic Bcl-2 antagonists.

## MATERIALS AND METHODS

### Cell lines and culture conditions

Prostate cancer cell lines, PC3, DU145 and LNCaP were cultured in 75cm² flasks with RPMI 1640 (Hyclone, SH 30096.01) enriched with 10% Fetal Bovine Serum (Gibco, 10270-196), Glutamine (2mM, Gibco, 25030-024) Penicillin and Streptomycin (50μg/ml) (Gibco, 5140-122). MEF cells were cultured in 75cm² flasks with high glucose DMEM (Hyclone, SH30285.01), 10% Fetal Bovine Serum (Hyclone), Glutamine (2mM) Penicillin and Streptomycin (50μg/ml) (Gibco). Cells were kept at 37°C in a humidified air incubator and 5% CO_2_. All cell lines were obtained from American Type Culture Collection (ATCC, USA) and have been verified by LGC standards and mycoplasma tested (Mycoplasma PCR detection kit, Minerva Biolabs, 11-1025). For the nutrient starvation experiments the cells were cultured in Earl's Balanced Salt Solution (EBSS) (Gibco, 24010-043) for 24h.

### Antibodies and reagents

The primary antibodies used in this study against cleaved caspase-7 (#9491), cleaved-PARP (#9541), phospho-Akt (Ser473) (#9275), Akt (#9272), Atg5 (#2630), Atg7 (#8558), LC3 I/II (#2775), cytochrome c (#12963), Beclin1 (#3738), p62 (#7695), were obtained from Cell Signaling Technology, p62 and GAPDH (Ab9485) from Abcam, p62 from Abnova, Bim (AAP-330) from Stressgen, Bcl-xL (610212) from BD Transduction Laboratories, β-actin (A5441) and cIAP (SAB3500268) from Sigma Aldrich, Bak, Bax (554104), Bcl-2 (554160) and RIPK1 (610459) from BD Europe; AIF (sc-9416), ULK1 (sc-33182), FADD (sc-271748), from Santa Cruz Biotechnology; Vps34 (38-2100) from Invitrogen.

Pancaspase inhibitor z-VAD-FMK (z-Val-Ala-Asp(OMe)-FMK) (FK009), Z-LEHD-FMK (Z-Leu-Glu(OMe)-His-Asp(OMe)-FMK) (FK022) from MP Biomedicals used at 10 μM, Rapamycin (R8781) used at 1 μM, Bafilomycin A1 (Sigma-Aldrich, B1793) used at 10 nM, Chloroquine (PHR1258) used at 50 μM, LY294002 (Sigma-Aldrich, L9908) used at 10 *μ*M, Necrostatin-1 (Sigma-Aldrich, N9037) used at 50 *μ*M, and Oligomycin A at 2.5 μg/ml (Sigma-Aldrich, 75351).

Sorafenib was provided by Bayer HealthCare Pharmaceuticals, Inc. In all the experiments, 20 μM sorafenib was used unless stated otherwise. Control samples were treated with the equimolar concentration of the appropriate vehicle.

### Constructs and transfections

Transfection with plasmids and siRNA experiments were performed according to protocols provided by Invitrogen or Polyplus transfection^TM^. Oligofectamine (Invitrogen) or INTERFERin^®^ (Polypus transfection^TM^) was used for transfections of siGFP (Silence^®^, AM4626, Ambion) siScramble (Cell signaling, #6568), siULK1 (5´ AGA AGA ACC UCG CCA AGU CTT 3´), sip62 (Cell signaling, 63995), siRIPK1 (ON-TARGETplus RIPK1 siRNA, Dharmacon, USA), siATG7 (Dharmacon, USA). Lipofectamine 2000 (Invitrogen) was used for all transfections with Atg5 (pCI-neo-hApg5-HA, Addgene, MA, USA), GFP-LC3, GFP-RFP-LC3, shBeclin (MISSION^®^ shRNA Plasmid DNA, Reference Sequence: NM_003766, Invitrogen). shScr (MISSION^®^ shRNA Control Vector, Invitrogen) was used as a control for the shBeclin1 transfections.

### Assessment of cell death by flow cytometry

Redistribution of plasma membrane phosphatidylserine is a marker of apoptosis and was assessed by Annexin V fluorescein isothiocyanate (FLUOS) (Roche, 14461000) [45, 46]. Briefly, 2 × 10^5^ cells per sample were collected, washed in PBS, pelleted, and re-suspended in incubation buffer (10 mM HEPES/NaOH, pH 7.4, 140 mM NaCl, 5 mM CaCl_2_) containing 1% Annexin V and PI. Samples were kept in the dark and incubated for 10 minutes prior to addition of another 400 μl of incubation buffer and subsequent analysis on Calibur flow cytometer (Becton Dickinson, San José, CA, USA) using Cell Quest software.

To detect sorafenib-induced changes in mitochondrial membrane potential, cell were stained with tetramethylrhodamine ethyl ester perchlorate (TMRE; Molecular Probes Inc. T699) as previously described [47]. Briefly, 1 nM TMRE was added to 10^6^ cells and incubated for 30 minutes. After washing the cells in PBS and TMRE, they were incubated for 10 minutes in the dark in 100 μl of incubation buffer (10 mM HEPES/NaOH, pH 7.4, 140 mM NaCl, 5 mM CaC1_2_, 25 nM TMRE) containing 1% Annexin V FLUOS. Prior to flow cytometric analysis, another 400 μl of incubation buffer was added. Staining was detected by FACS Calibur flow cytometer (Becton Dickinson, San José, CA, USA) and the data analysed with the CellQuest software, provided by Becton Dickinson.

### Transmission and immuno-electron microscopy

DU145 cells were incubated with 20 μM sorafenib for 12 and 24 h. After the indicated times, cells were harvested and processed as described [48]. Micrographs were taken at 2000-2500x magnification. Ultrathin sections were incubated in 8% H_2_O_2_, washed in dH_2_O and incubated in 0.01M PBS pH7.4 and blocked with 5% goat serum (X0907, Dako, Denmark) in PBS for 30 minutes. Sections were then incubated with LC3 mouse monoclonal antibody (MBL, M152-3) in a dilution of 1:10 in PBS containing 1% BSA for 20h at 4°C. Subsequently sections were incubated in 0.05M Tris/HCl pH7.4, 0.05M Tris/HCl pH7.2 : 0.2% BSA (1:1) and finally in 0.05M Tris/HCl pH8.2 : 1% BSA (1:1) followed by incubation with 10nm colloidal gold conjugated secondary antibody in a dilution 1:10 for 1h at RT. They were then washed in 0.05M Tris/HCl pH7.2 : 0.2% BSA (1:1), 0.05M Tris/HCl pH7.4 and dH_2_O and dried on blotting paper and stained in 7.5% Uranyl Acetate ethanol solution and then in 0.4% Lead Citrate aqueous solution at RT. Sections were visualized and micrographs were captured on a FEI Morgani 268 Transmission Electron Microscope.

### Mitochondrial respiration

Mitochondrial oxygen consumption of DU145 cells treated for the indicated time points with 20 μM sorafenib was monitored with the oxygen-sensitive electrode (Hansatech Instruments, Norfolk, UK) and analyzed with the Oxygraph Plus software (Hansatech Instruments). Cells (4 × 10^6^) were harvested, and resuspended in 300 μl of medium. After closing the lid, mitochondrial State 3 respiration was monitored for 3-4 minutes. State 4 respiration was attained after addition of oligomycin (2.5 μg/ml), an inhibitor of mitochondrial ATP synthase. The maximum activity of the respiratory chain (uncoupled respiration) was assessed after addition of a protonophore, carbonyl cyanide m-chlorophenylhydrazone (CCCP 5 μM).

### ATP measurements

For ATP content determination DU145 cells were harvested, collected and lysed using ATP releasing buffer (Sigma). The concentration of ATP was quantified by using the ATP Bioluminescent Assay Kit (Sigma, FLAA-1KT) according to the manufacturer's instructions.

### Immunocytochemistry and time lapse microscopy

The cytotoxic effects of sorafenib on DU145 cell lines was analysed by staining of mitochondria with MitoTracker (Mol. Probes, Inc., M7514) and co-staining with an antibody against cytochrome c as previously described [49]. Briefly, for mitochondrial staining, cells were cultured in 12-well plate containing sterile cover slips for 24h, just before staining they were incubated for 30 minutes in normal growth medium containing 5 μm MitoTracker (Mol. Probes, Inc.) and fixed in 4% paraformaldehyde (PFA) for 20 min, permeabilized using digitonin (10 μg/ml) diluted in PBS for 10 minutes and stained with anti-cytochrome c antibodies for 1h at room temperature, followed by rabbit-anti-mouse FITC-conjugated antibodies (DAKO, F0261). For the intracellular stainings of p62 (mouse) and RIPK1 (rabbit), cells were fixed with PFA 4% PFA and permeabilised with digitonin for 10 min. The cells were then stained with anti-rabbit Alexa 488 and anti-mouse 594. The images were recorded on a DAS Leitz DM RB microscope with a Hamamatsu C4880 dual-mode cooled CCD camera and further processed using Photoshop software (Adobe). Time lapse confocal microscope was performed. For the time lapse confocal microscopy, DU145 cells stably transfected with GFP-LC3 were stained with mitotracker as aforementioned and placed in the confocal microscope time-lapse with heated chamber, lense, stage and provision of 5% CO. Images were captured every hour for 24h.

### Western blot analysis

Cells were harvested and homogenized in RIPA lysis buffer (10 mM Tris, pH 7.2, 150 mM NaCl, 1% deoxycholate, 1% Triton, 0.1% SDS, 5 mM EDTA) containing complete protease inhibitor cocktail, phospho-stop, (Roche Diagnostics, Meylan, France), dithiothreitol (Sigma Aldrich, D0632) and vanadate (Life Technologies, S5608). After 30minutes on ice, samples were sonicated and protein quantification was carried out using a Bio-Rad Bradford protein assay. Equal amounts of soluble proteins (15-25 μg) were loaded with LDS sample buffer (NuPAGE, Life Technologies) and dithiothreitol denaturated by boiling and resolved by sodium dodecyl sulphate-polyacrylamide gel electrophoresis (SDS-PAGE) and transferred in 20% methanol to a methanol-activated PVDF membrane (Perkin Elmer, NEF1002001PK). After blocking in 5% non-fat dry milk in TBS for 1h and probing with a specific primary antibody overnight and a horseradish peroxidase-conjugated secondary antibody for 1h, both in 5% non-fat dry milk in TBS, the protein bands were detected by Western Lightning (Perkin Elmer) and X-ray film exposure (Kodak). Protein loading was normalized by using anti-GAPDH or anti-actin antibodies.

### Assessment of autophagy

For the GFP-LC3 experiments, DU145 cells were transfected by 4μg of pEGFP-LC3 plasmid using Lipofectamine 2000 (Invitrogen, 11668-019) based on manufacturer's recommendation [50]. Twenty four hours after transfection the growth media was replaced by complete RPMI 1640 supplemented with 1 mg/ml G418 (Sigma) for selection. EGFP-LC3 positive cells were selected by MoFlo™ XDP Cell Sorter (Beckman Coulter) and cultured for 3 weeks under G418 selection pressure. Following treatment, the cells were fixed with 4% PFA and mounted using Vectashield with DAPI. The images were recorded on a Zeiss Axioplan-2 microscope with a Zeiss dual mode cooled CCD camera and Axiovision software 4.1.

### Long lived protein degradation assay

The Click-iT^®^ AHA Alexa Fluor^®^ 488 Protein Synthesis HCS Assay (L-Azidohomoalanine) (Invitrogen, C10289) kit was used, as previously described [32]. Briefly, 20,000 cells were plated in a 96-well plate overnight. The cells were washed with PBS and incubated with L-methionine free medium (Gibco, 21013-024) for 30 minutes. Afterwards, Click-iT^®^ AHA (L-azidohomoalanine) reagent (component A) was added for 18 hours. The cells were washed with complete DMEM medium for 2 hours, in order to chase out short lived proteins and were treated with the indicated drugs for 24 hours. The cells were washed with PBS followed by fixation with 4% PFA for 15 minutes. The cells were washed with 3% BSA and permeabilised with digitonin (10 μg/ml) for 20 minutes. Next, the cells were washed again with 3% BSA and received Click-iT^®^ AHA supermix (Component B) and Click-iT^®^ AHA buffer additive (Component C) for 2 hours, followed by a wash with 3% BSA. The cells were with intracellular fluorescent proteins were measured by flow cytometry and the data analysed with the CellQuest software, provided by Becton Dickinson.

### Immunoprecipitation

For immunoprecipitation, μMACS™ Protein A/G MicroBeads MultiMACS™ Protein A/G Kit (Miltenyi Biotec, 130-071-001) was used. Briefly DU145 cells treated with 20 μM sorafenib for 24hs, lysed with lysis buffer (150 mM NaCl, 1% Triton X-100, 50 mM Tris HCl (pH 8.0)) and 200 μg protein pre -washed with 100 μl of microbeads for 1 hour and beads were removed. Supernatant were incubated with 100 μl of pre-washed beads and 1 mg of RIPK antibody overnight at 4°C then beads were washed using MACS separation columns. 30 μl of separated protein were run on a SDS-PAGE gel and 20 μg of cell lysate run as input.

### Proximity ligation assay (PLA)

For the proximity ligation assay, the Duolink^®^ In Situ Detection Reagents Red (Sigma, DU092008) to was used according to the manufacturer's protocol. Briefly, the DU145 cells were cultured on cover slips, treated with 20 μM sorafenib for 24h, fixed with 4% PFA and then permeabilized using digitonin as described above. RIPK (mouse) and p62 (rabbit) antibody were used as primary antibody. After 1h incubation, cells were washed twice and PLA probe MINUS and PLA probe PLUS were added and incubated for 1h at 37° C. After washing, ligation reaction was done for 30 minutes and the amplification was run for 100 minutes. Then the cells were washed, mounted and captured by using the LSM 510; Carl Zeiss confocal microscope. The Zeiss AIM user interface software was used to process the images to a two-dimensional illustration of the summary of all dots in each cell Zeiss 550 confocal microscope.

### Clonogenic assays

One hundred DU145 cells were plated and cell transfected with either siScr or siRNA against p62. After 24h cells were treated by 50 μM Necrostatin-1 and/or 20 μM sorafenib for 24h. After 10 days the clones were fixed, stained with crystal violet and counted.

### Prostate cancer tissue microarray

A tissue microarray (TMA) was constructed from a series of 41 radical prostatectomy specimens collected in 2003 at Karolinska Hospital, Solna, Sweden. The patients underwent surgery for primary adenocarcinoma of the prostate. None of them had received hormonal therapy or radiotherapy, prior to surgery. After fixation, the prostate was inked, sliced horizontally at 4 mm and totally embedded. The specimens were dehydrated, paraffin embedded, cut into 4 μm sections and stained with hematoxylin and eosin. The TMA was constructed using a Beecher, Manual Arrayer I (Beecher Instruments Inc, Sun Prairie, WI, USA). A representative tumor core with a diameter of 1 mm was collected from the main tumor of each specimen and another core was taken from benign tissue, usually within benign prostatic hyperplasia in the transition zone. Intensity and extent of immunoreactivity and their product (IR) were evaluated in each core by a trained pathologist (LE). The material was evaluated at two occasions with a two-week interval. The intensity was scored from 0 (no staining) to 3 (most intense staining) based on the strongest staining of the core. The extent of positive intracytoplasmic staining was evaluated in a semiquantitative manner. Scoring was based on percentage of stained epithelial cells and graded from 1 to 3, signifying 1-33%, 34-66%, and > 66%, respectively.

### Statistical analysis

The statistical analysis for paired data was performed by Student's *t* test. *P*-values < 0.05 were considered significant. All reported *p*-values are two-sided.

## SUPPLEMENTARY MATERIAL FIGURES AND VIDEOS






