# Supplementary material for: Sorafenib-induced defective autophagy promotes cell death by necroptosis
Source: Oncotarget. 2015 Sep 22;6(35):37066–82. doi: 10.18632/oncotarget.5797 (PMC4741916; doi:10.18632/oncotarget.5797)
Supplement: Supplementary file 1 [file oncotarget-06-37066-s001.pdf]

Sorafenib-induced defective autophagy promotes cell death by necroptosis

Supplementary Material

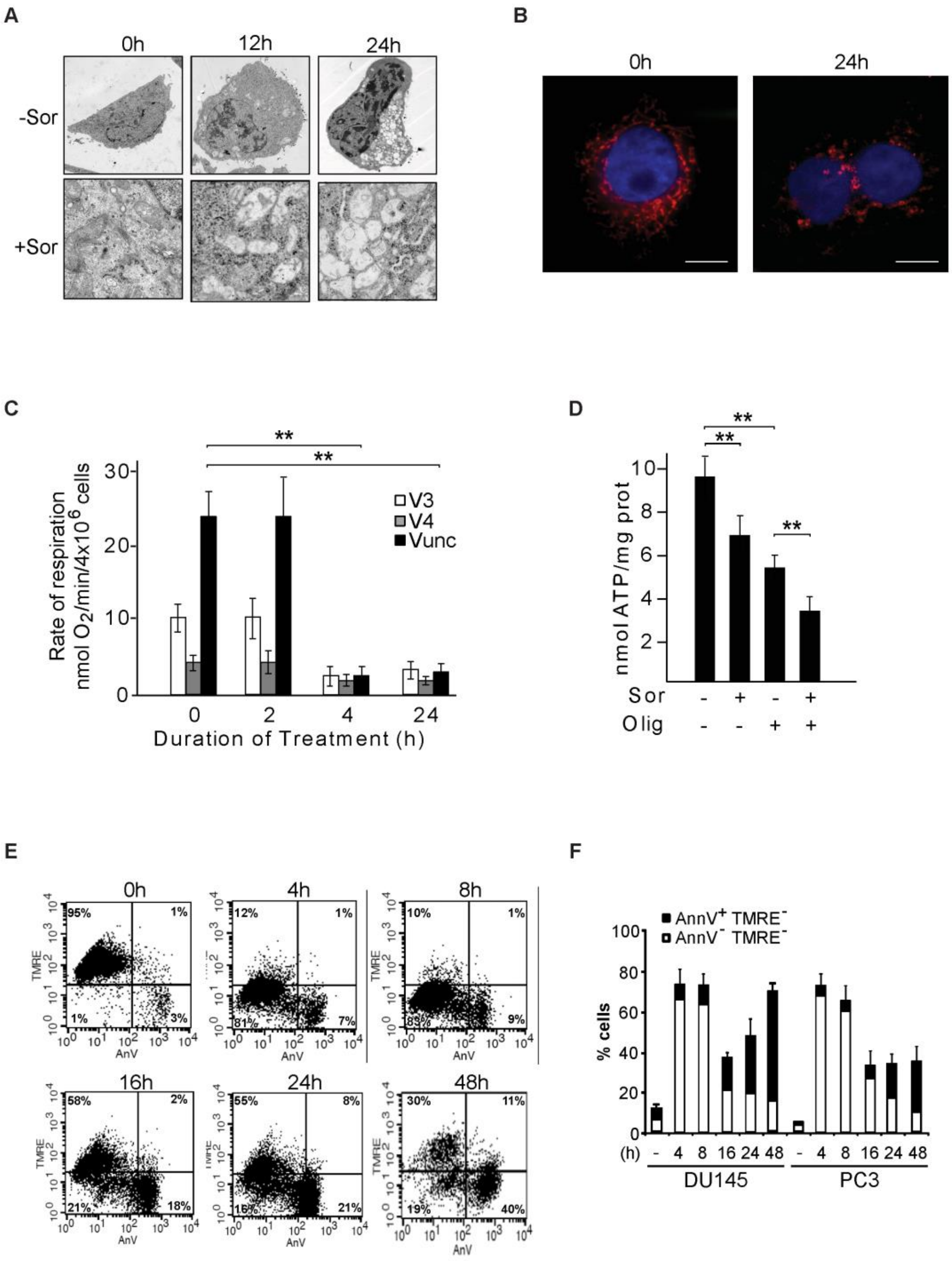

Supplementary figure 1. (A) Transmission electron microscopy of DU145 cells treated for the indicated time points with 20 µM Sor for the indicated time points (scale bar: 500 nm); (B) Immunocytochemistry staining with Mitotracker of DU145 cells treated with 20 µM Sor for 24h (Scale bar: 1 µm); (C) Mitochondrial oxygen consumption of DU145 cells treated for the indicated time points in DU145 cells treated with 20 µM Sor (means ± SD, n≥3, **<0.01); (D) Quantification of intracellular ATP concentration in DU145 cells treated with oligomycin (2.5 µg/ml) in the presence or absence of 20 µM Sor, 48h (means ± SD, n=3, **<0.01); (E) Dot plots of DU145 cells treated with 20 µM Sor for the indicated time points followed by staining with Annexin V (AnnV)/TMRE and measurement by flow cytometry; (F) Quantitative analysis of AnnV/TMRE positive DU145 cells treated with 20 µM Sor for the indicated time points, (means ± SD, n≥3); (E)

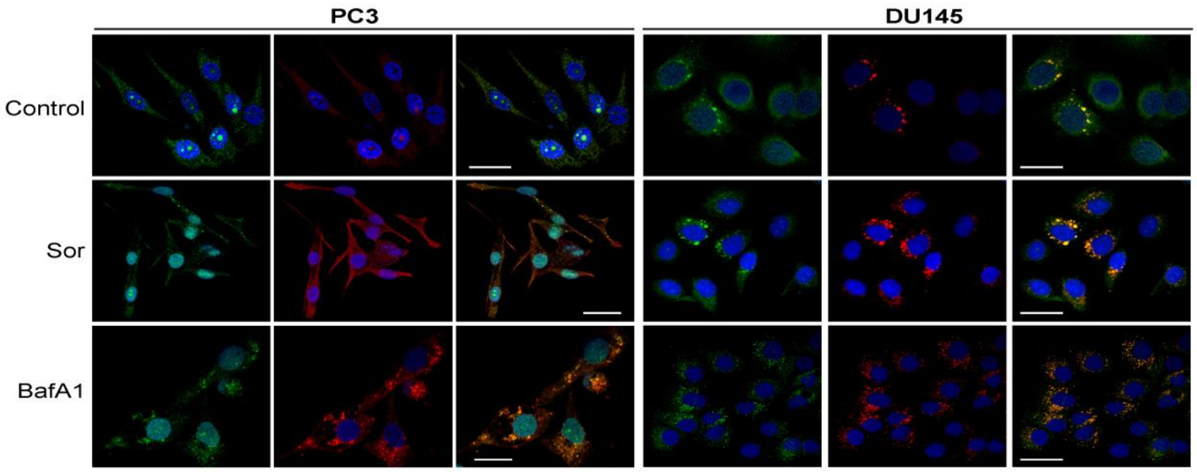

Supplementary figure 2. Confocal microscopy imaging of PC3 and DU145 cells treated with 20µM Sor or 10 nM BafA1 for 24h and stained for endogenous LC3 (Alexa 488) and p62 (Alexa 594) (Scale bar: 2 µm, means ± SD, n≥3, **<0.01)

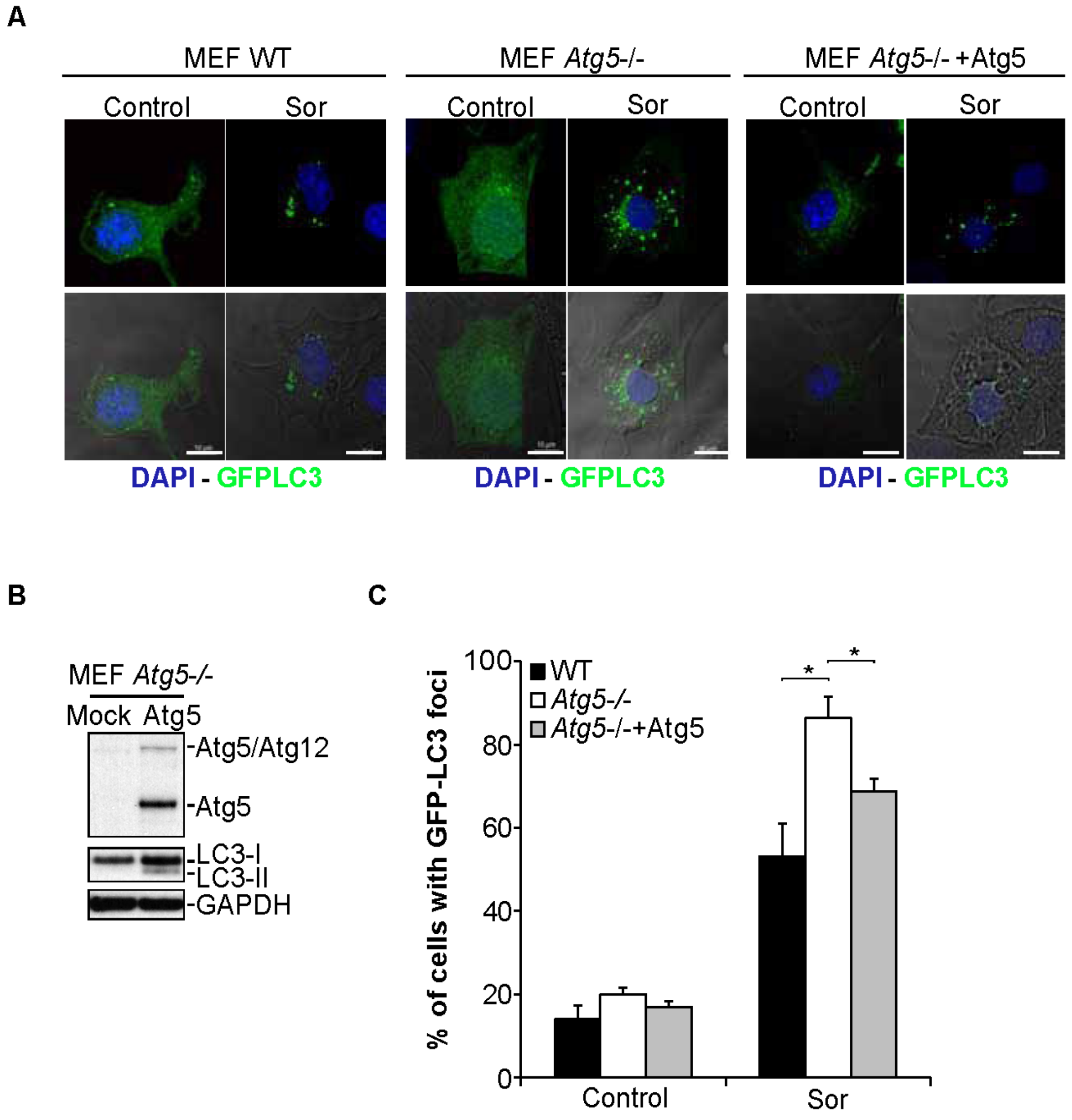

Supplementary figure 3. Confocal microscopy imaging of the indicated MEF cells treated with 20µM Sor or 10 nM BafA1 for 24h, (Scale bar: 2 µm); (B) Western blot analysis of the indicated proteins in *Atg5-/-* MEF cells transiently transfected with a pCDNA plasmid or Atg5; (C) Quantification of the indicated MEF cells with GFP-LC3 positive foci after treatment with either 20 µM Sor for 24h (200 cells counted, n=2, *<0.05).

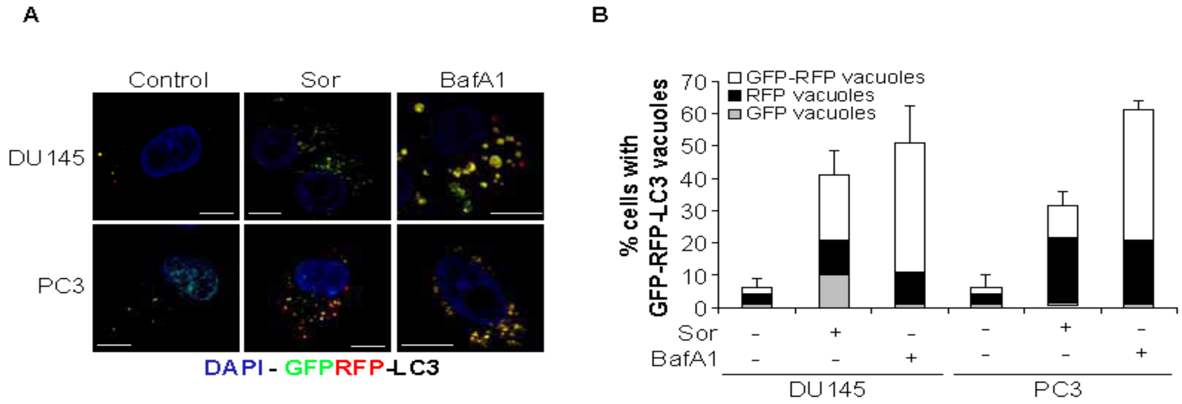

Supplementary figure 4. (A) Confocal microscopy imaging of DU145 cells stably transfected with GFP-RFP-LC3 and treated with 20μM Sor or 10 nM BafA1 for 24h. The percentage of cells with fluorescent puncta was counted (200 cells, n=2).

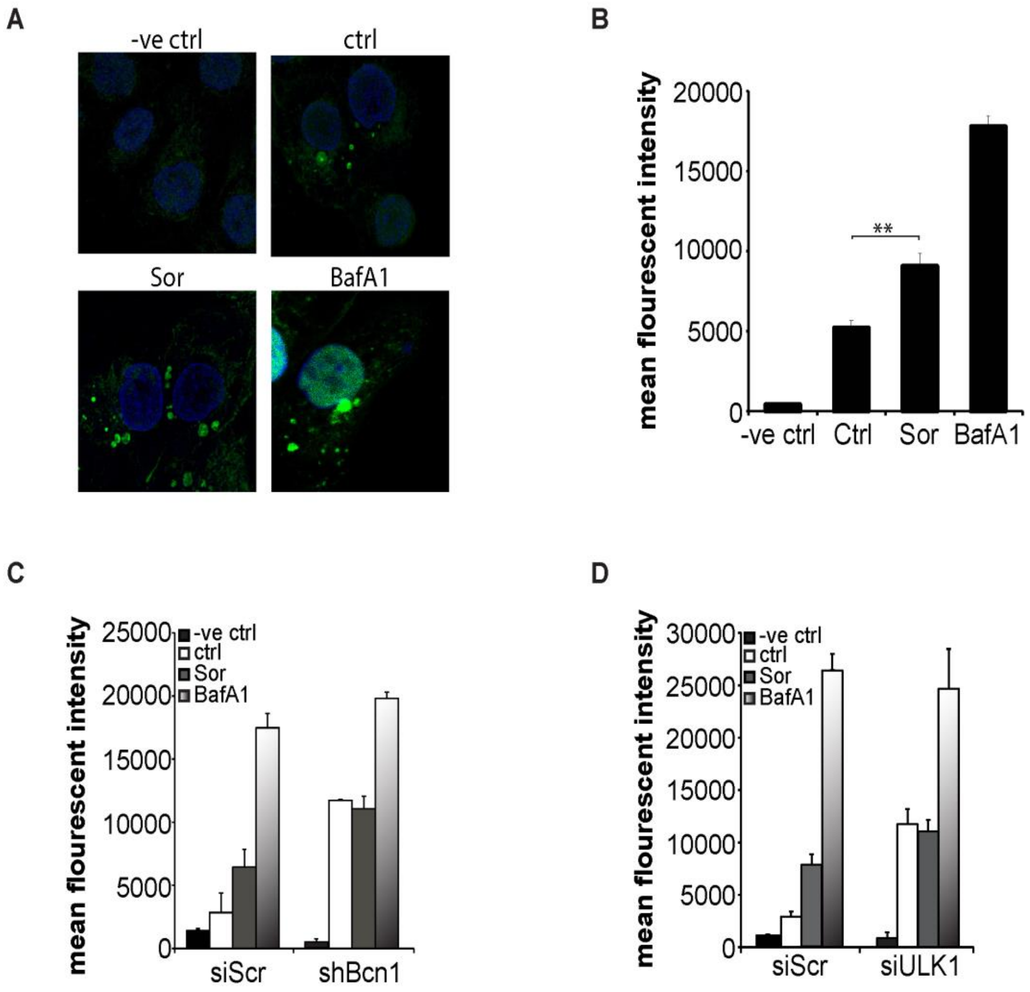

Supplementary figure 5. (A) Confocal microscopy imaging of DU145 cells stained with fluorescent methionine analogue (AHA) as described in the materials and methods, treated with 20µM Sor or 10 nM BafA1 for 24h (representative images of two independent experiments); (B) Quantification by flow cytometry of the AHA-positive green fluorescent DU145 cells treated with 20µM Sor or 10 nM BafA1 for 24h (means ± SD, n=3, **<0.01); (C) Quantification by flow cytometry of the AHA-positive green fluorescent DU145 shScramble and shBeclin1 cells treated with 20µM Sor or 10 nM BafA1 for 24h (means ± SD, n=2); (D) Quantification by flow cytometry of the AHA-positive green fluorescent in DU145 siScramble and siULK1 cells treated with 20µM Sor or 10 nM BafA1 for 24h (means ± SD, n=2).

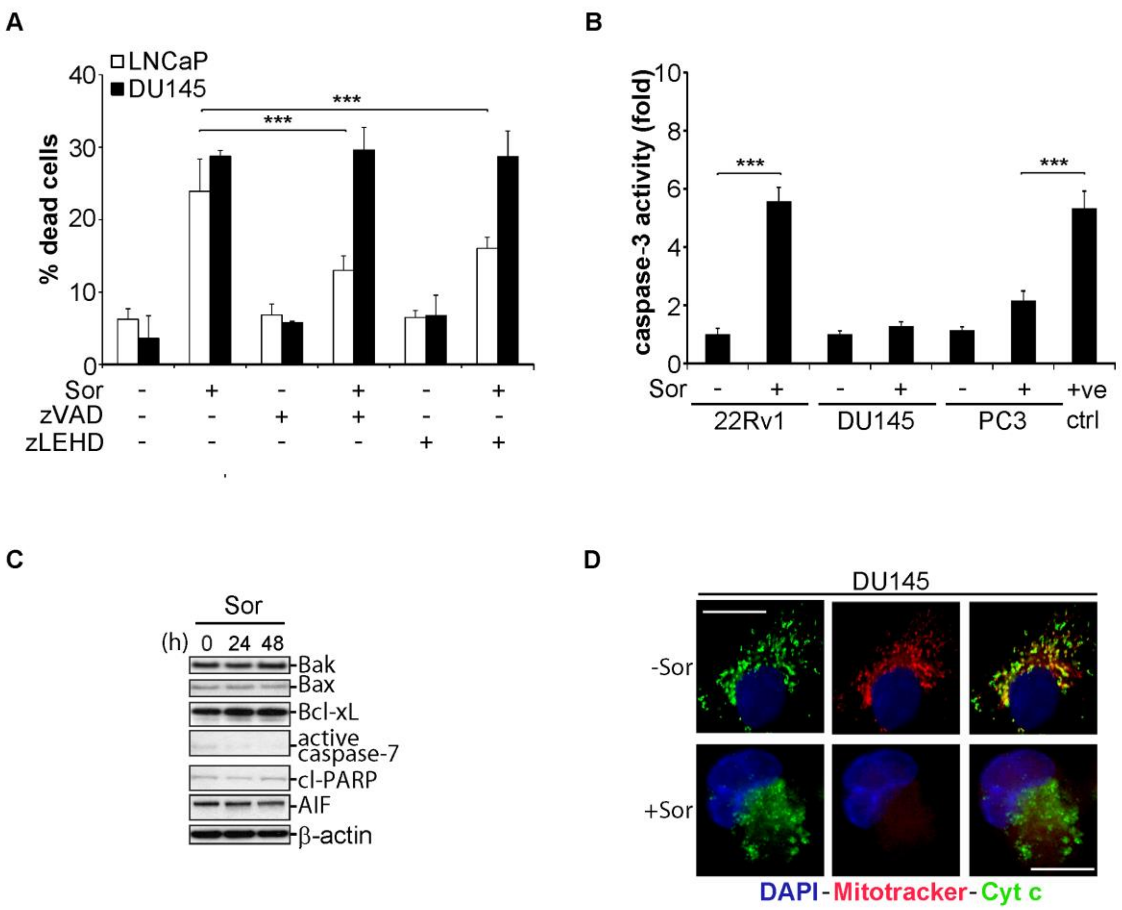

Supplementary figure 6. (A) Quantification of cell death in LNCaP and DU145 cells pre-treated with zVAD.fmk or zLEHD.fmk inhibitor followed by 20 µM Sor for 24h; (B) Measurement of caspase-3 activity in the indicated prostate cancer cell lines after treatment with 20 µM Sor for 24h; (C) Western blot of the indicated proteins in DU145 treated with 20 µM Sor for 24h and 48h; (D) Immunoflourescent staining of mitochondrial membrane potential (appearing in red, mitotracker) and cytochrome c (appearing in green, FITC) in DU145 cells treated with 20 µM Sor for 24h (scale bar = 10 µm).

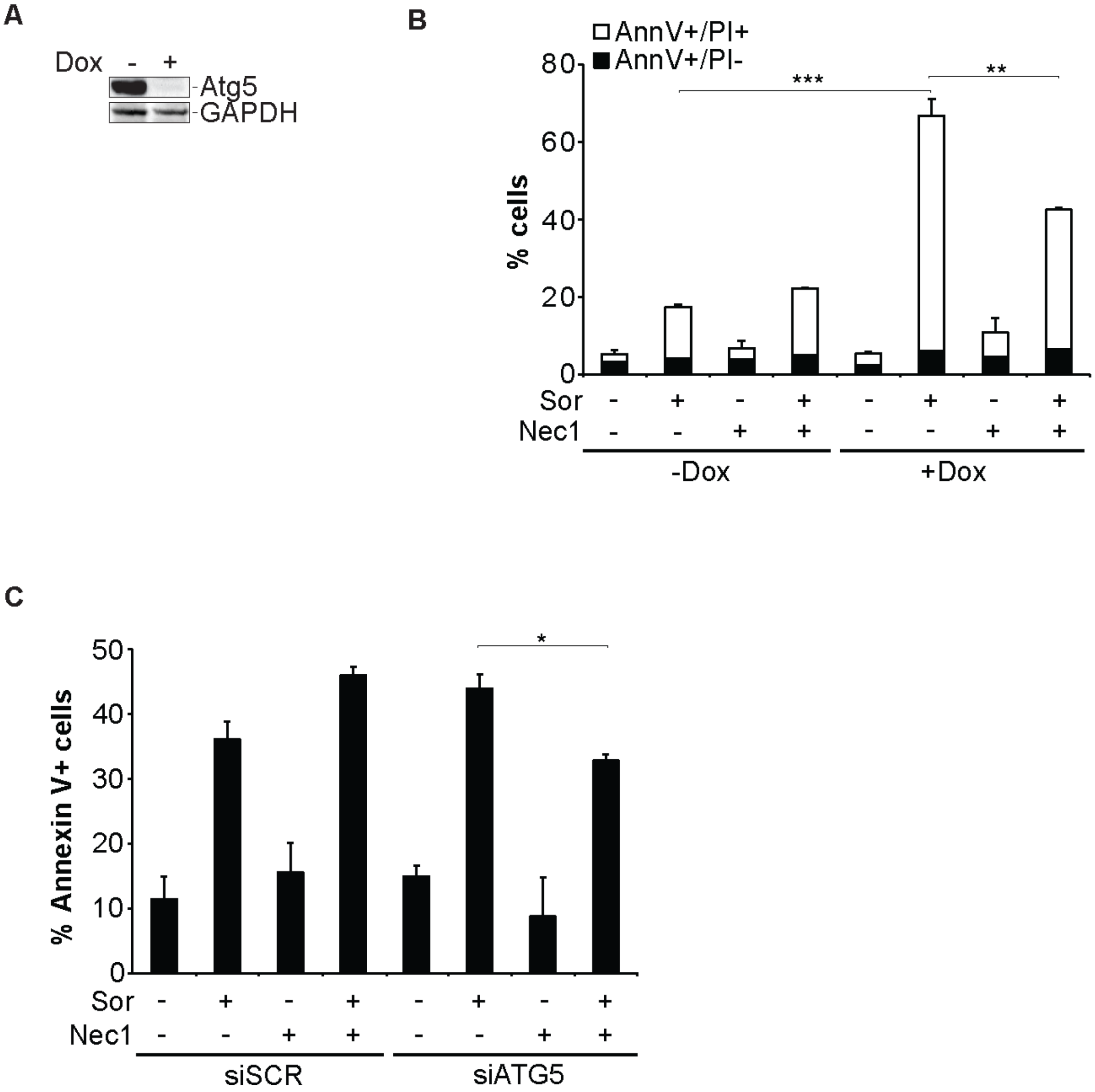

Supplementary figure 7. (A) Western blot of Atg5 in *Atg5-/-* MEF cells stably transfected with Atg5 with a tet-off promoter treated with 10 ng/ml doxycycline (Dox); (B) Quantitative analysis of Annexin V/PI positive *Atg5-/-* MEF cells stably transfected with Atg5 with a tet-off promoter and cultured in 10 ng/ml doxycycline (Dox), pre-treated with 50 µM Nec-1 followed by 20 µM Sor for 48h means ± SD, n=3, **<0.01, ***<0.005); (C) Quantitative analysis of Annexin V positive PC3 cells transiently transfected with siRNA against Atg5 and pre-treated with 50 µM Nec-1 followed by 20 µM Sor for 48h (means ± SD, n=3, *<0.05.

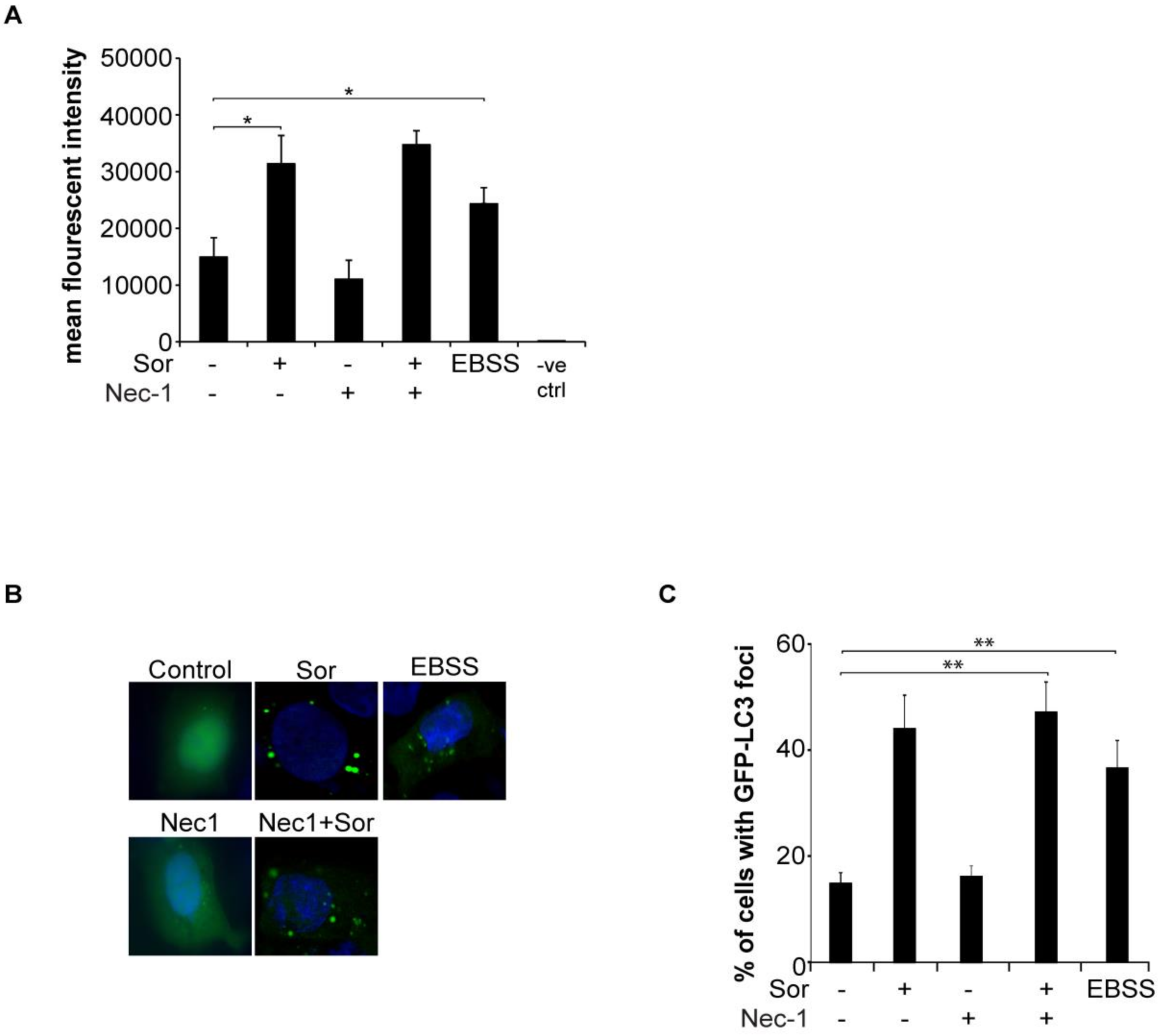

Supplementary figure 8. (A) Quantification by flow cytometry of the AHA-positive green fluorescent DU145 cells treated with 50 µM Nec-1 followed by 20µM Sor 24h (means ± SD, n=3, *<0.05); (B-C) Confocal microscopy imaging and quantification of DU145 cells stably transfected with GFP-LC3 and treated either with EBSS or with 50 µM Nec-1 followed by 20µM Sor 24h (200 cells counted in each slide, means ± SD, n=3, **<0.01).

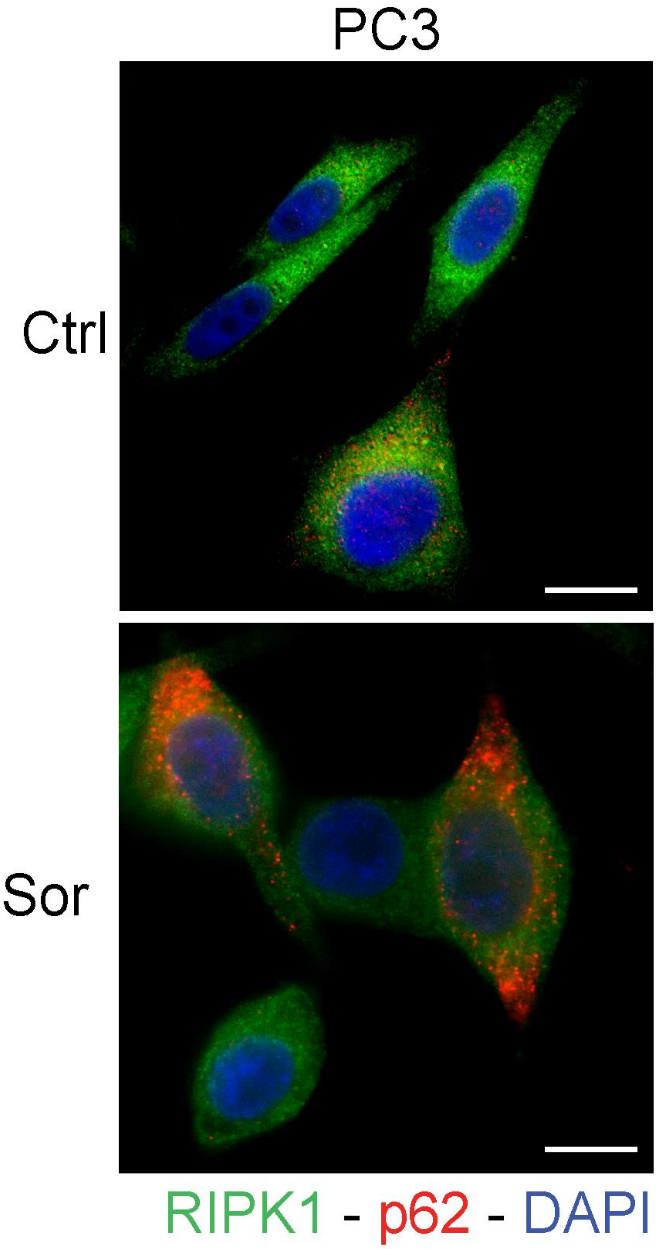

Supplementary figure 9. Representative confocal microscopy images of PC3 cells stained for p62 and RIPK1 after treatment with 20μM Sor for 24h (Scale bar: 2 μm).
